# Epigenetic modifications evidenced by isolation of proteins on nascent DNA and immunofluorescence in hydroxyurea-treated root meristem cells of *Vicia faba*

**DOI:** 10.1007/s00425-023-04249-2

**Published:** 2023-10-09

**Authors:** Aneta Żabka, Natalia Gocek, Justyna Teresa Polit, Janusz Maszewski

**Affiliations:** Faculty of Biology and Environmental Protection Department of Cytophysiology, University of Lodz, 90-236 Lodz, Poland

**Keywords:** DNA replication, Epigenetic modifications, Hydroxyurea, Isolation of proteins on nascent DNA (iPOND), Replication stress, *Vicia faba*

## Abstract

**Main conclusion:**

By implementation of the iPOND technique for plant material, changes in posttranslational modifications of histones were identified in hydroxyurea-treated root meristem cells of *Vicia*.

**Abstract:**

Replication stress (RS) disrupts or inhibits replication forks and by altering epigenetic information of the newly formed chromatin can affect gene regulation and/or spatial organisation of DNA. Experiments on *Vicia faba* root meristem cells exposed to short-term treatment with 3 mM hydroxyurea (HU, an inhibitor of DNA replication) were aimed to understand epigenetic changes related to RS. To achieve this, the following histone modifications were studied using isolation of proteins on nascent DNA (iPOND) technique (for the first time on plant material) combined with immunofluorescence labeling: (i) acetylation of histone H3 at lysine 56 (H3K56Ac), (ii) acetylation of histone H4 at Lys 5 (H4K5Ac), and (iii) phosphorylation of histone H3 at threonine 45 (H3T45Ph). Certainly, the implementation of the iPOND method for plants may prove to be a key step for a more in-depth understanding of the cell's response to RS at the chromatin level.

## Introduction

More than animals, plants depend on the local environment, suggesting that they have evolved a vast number of distinctive and efficient biochemical mechanisms to control DNA replication, cell divisions, and differentiation, all these processes being targeted precisely for successful progression of their specific developmental traits. To prevent the influence of adverse factors or threatening conditions, plants have advanced an intricate and complex molecular system that can recognize stress and elicit appropriate response activities (Bej and Basak 2017). Accordingly, they trigger various signal transduction pathways, switch on stress-inducible genes, cell cycle checkpoints and induce adequate changes at the biochemical, physiological and morphological levels. In consequence, they stimulate DNA damage repair machineries, temporal metabolic dormancy, or an animal-like programmed cell death (AL-PCD) processes (Hirayama and Shinozaki 2010; Viggiano and de Pinto 2017; Żabka et al. 2017).

Due to the sessile lifestyle, plants are continuously exposed to various environmental stresses (biotic and abiotic), some of which can cause DNA damage. Accordingly, they must integrate developmental processes and environmental signals (including poor resource availability, unfavorable water, soil, and light conditions), and to coordinate cell division, cell differentiation and organ growth (Nikitaki et al. 2018; Gentric et al. 2021; Szurman-Zubrzycka et al. 2023).

Plants, in common with all eukaryotes, organize their DNA into multiple units termed replicons, each containing a single replication origin (ORI). As a result, there are thousands of sites scattered across the genome in which DNA biosynthesis initiates (Bryant and Aves 2011; Leonard and Méchali 2013). The principal feature of this process is that replicons function in groups and the timing of their sequential activation during S-phase, at least in part, depends on the chromatin structure in which ORIs reside. Early replication of chromosomal domains, enriched in ORIs and origin recognition complexes (ORCs; multi-subunit DNA-binding protein complexes) are generally observed in transcriptionally operative gene-rich domains with active epigenetic marks. On the contrary, late replicating domains are observed in origin-poor heterochromatin regions that have low gene density, generally associated with repressive epigenetic states and numerous transposon elements (Fragkos et al. 2015). Consequently: (1) a precise temporal pattern of DNA replication occurs at a limited number of discrete foci localized within the nucleus, (2) not all ORIs fire at the same time, and (3), synchronously firing ORIs are not homogenously distributed throughout the genome. All this results in the appearance of well-conserved early and late replication pattern during S-phase progression, which can be visualized at the fluorescence microscopy level (Casas-Delucchi and Cardoso 2011).

Similar to all other organisms, plant DNA molecules need to strike a balance: on the one hand, they have to be rigid enough to protect the genetic information and to maintain chromosomal integrity, and on the other, they must be available and accessible to the cell cycle regulated activities and molecular interactions indispensable for differentiation-associated processes (Inzé and De Veylder 2006). Accordingly, the process of replication must be tightly regulated by means of precise synchronization of cellular pathways engaged to ensure sufficient energy and material supply and, in case of potential emergency, to arrange functional repair of the genome. Dysfunction of these mechanisms may contribute to the development of replication stress (RS) (Nisa et al. 2019).

Our current study on *Vicia faba* root meristem cells aimed to investigate and update our understanding of DNA replication-associated epigenetic changes appearing after short-term exposure to 3 mM hydroxyurea (HU; a classical inhibitor of DNA replication; Koç et al. 2004). To achieve this, histone H3 and H4 modifications investigated by iPOND (isolation of proteins on nascent DNA) technique combined with immunocytochemical labeling included: (i) histone H3 acetylation at lysine 56 (H3K56Ac), involved in S-phase transition (Li et al. 2008), DNA damage response (Driscoll et al. 2007; Masumoto et al. 2005; Wurtele et al. 2012), and transcription (Xu et al. 2005; Rufiange et al. 2007); (ii) histone H4 acetylation on Lys 5 (H4K5Ac), showing a strong correlation with DNA replication (Jasencakova et al. 2000); (iii) histone H3 phosphorylation on threonine 45 (H3T45Ph), which is involved in DNA replication and apoptosis (Hurd et al. 2009; Baker et al. 2010).

For the first time, by implementation and adaptation of the iPOND method, which allows the 'tracking' of protein association and deposition in active and stalled or damaged replication forks (RFs; Sirbu et al. 2011; Olcina et al. 2016), it was possible to determine and to compare changes in posttranslational modifications of histones in the untreated (control) and HU-treated plant root meristem cells exposed to the DNA replication stress. To date, site-specific analysis of active and arrested replicons has only been performed on animal cells (e.g. Sirbu et al. 2011).

## Materials and methods

### Plant materials

Sterile seeds of faba bean (*Vicia faba* subsp. *minor* L.) were sown on dishes lined with moist filter paper and germinated in the dark at 20 °C. After 96 h, young seedlings with primary roots ranging from 1.5 to 2 cm were placed in Petri dishes containing distilled water (control) and 3 mM hydroxyurea solution (HU). Incubations were performed for 2 h in the dark. Primary root were chosen for practical reasons (large number of cells, short time of RAM isolation and low volume of incubation solutions).

### Feulgen staining

After incubation in water and HU, severed roots were cut off, fixed in ice-cold Carnoy’s solution (absolute ethanol and glacial acetic acid; 3:1, v/v) for 1 h, washed with ethanol, and then hydrolysed in 4 M HCl for 2 h. The pararosaniline (Schiff's reagent) staining procedure was performed according to the standard method (e.g. Żabka et al. 2010). After rinsing in SO_2_-water (3 times) and distilled water, root meristems were cut off, placed in 45% acetic acid, crushed on slides, dried and embedded in Canada balsam.

### EdU labeling

Control and HU-treated seedlings were incubated with 10 μM 5-ethinyl-2’-deoxyuridine (EdU, Thermo Fisher Scientific, Warsaw, Poland) for 20 min, in the dark. Excised root tips were fixed in PBS-buffered 4% paraformaldehyde (4 °C; pH 7.4) for 45 min and macerated for 15 min in citrate-buffered 2.5% pectinase (Sigma-Aldrich), pH 5.0. Meristems were crushed on slides (Polysine™, Menzel-Gläser, Germany), dried, and after washing with PBS buffer, replication of nuclear DNA was visualized using the Click-iT DNA Alexa Fluor® 555 Imaging Kit (Thermo Fisher Scientific), according to the included instructions. Cell nuclei were stained for 15 min using 15 μM 4’,6-diamidino-2-phenylindole (DAPI; Sigma-Aldrich). Dried slides were embedded in a mixture of PBS/glycerol/DABCO (2.3% diazabicyclo(2.2.2)octane).

### iPOND technique

Isolation of proteins on nascent DNA (iPOND) technique was performed according to Sirbu et al. (2011, 2013), with some modifications for plant material. About 1000 dissected root meristems of *V. faba* (corresponding approximately to 1.0 × 10^8^ cells) were labeled with EdU (10 min), transferred to 10 µM thymidine solution (Sigma-Aldrich) for 10 min, and finally exposed to H_2_O (control series) or 3 mM HU. After 2 h, roots were fixed with 1% formaldehyde (Sigma-Aldrich) in PBS and incubated for 20 min at room temperature (RT). Formaldehyde cross-linking was inhibited by addition of 1.25 M glycine (Sigma-Aldrich). After washing with PBS (3 times), root meristems were trimmed to the length of 1 mm (jointly producing about 1.0 × 10^8^ cells per series). Then, root tips were placed in a citric acid-buffered digestion solution (pH 5.0) containing 2.5% pectinase, 2.5% cellulase and 2.5% pectolyase (Sigma-Aldrich), and incubated at 37 °C for 60 min. After the digestion solution was removed, root tips were washed with PBS, and incubated for 30 min (RT) in permeabilization buffer (0.25% Triton X-100 in PBS). After centrifugation for 5 min at 900 g (4 °C), the supernatant was carefully decanted. Cells were washed 2 times with 0.5% BSA in PBS, centrifuged for 5 min at 900 g, (4 °C). The obtained pellet was placed into a click reaction cocktail (on ice, under dark conditions) consisting of: 1 × PBS (4.35 ml), 10 µM biotin-azide (0.05 ml; Invitrogen), 10 mM sodium ascorbate (0.5; Sigma-Aldrich), and 2 mM CuSO_4_ (0.1 ml). Incubation with the cocktail was performed by gentle rotation at RT for 1–2 h. After centrifugation for 5 min at 900 g (4 °C), the supernatant was removed. Cells were washed with cold 0.5% BSA dissolved in PBS, centrifuged for 5 min at 900 g (4 °C), washed with PBS and decanted. Lysis buffer (1% SDS in 50 mM Tris, pH 8.0) was supplemented by aprotinin (Sigma-Aldrich) and leupeptin (Sigma-Aldrich) at a concentration of 1 μg/ml (each) before use. After resuspending the samples in lysis buffer, cells were sonicated (13–16 W, 20 s constant pulse, 40 s cell lysate pause; total pulse time: 4–5 min per sample). Then, samples were centrifuged for 10 min at 16 000 g, at RT. The resulting lysate was diluted 1:1 (v/v) with cold PBS containing 1 μg/ml of aprotinin and 1 μg/ml of leupeptin. 15 μl of lysate was placed on ice and kept as an input sample. The remaining lysate was used for streptavidin capture of biotin-labeled nascent DNA. Each experimental sample was incubated with streptavidin agarose beads at a concentration of 100 μl of bead suspension per 1 × 10^8^ cells. First, the bead suspension was centrifuged at 1800 g for 1 min at RT, washed 2 times with lysis buffer containing aprotinin and leupeptin, and the supernatant was aspirated after each wash. Finally, the beads were washed again in PBS containing aprotinin and leupeptin (1:1 beads:PBS), the supernatant was aspirated and the beads were resuspended in PBS containing protease inhibitors. An equal volume of biotin beads was added to each sample and mixed for 16–20 h (4 °C). On the following day, streptavidin-agarose beads with captured DNA and associated proteins were centrifuged for 3 min at 1800 g, at RT. Most of the supernatant was withdrawn very slowly and carefully. 1 ml of cold lysis buffer (no additives) was added, spun at RT for 5 min, centrifuged for 1 min at 1800 g (RT), and the supernatant was drawn away. The beads were washed 1 time with 1 ml of 1 M NaCl, rotated at RT for 5 min, centrifuged for 1 min at 1800 g (RT), carefully aspirated and the supernatant discarded. Cold lysis buffer (no additives) was added to wash the beads, rotated at RT for 5 min, centrifuged for 1 min at 1800 g, at RT, carefully aspirated and the supernatant discarded. After the last wash, the entire supernatant was aspirated. To elute proteins bound to nascent DNA, 2 × SDS Laemmli sample buffer (2 × SB; 0.4 g SDS, 2 ml 100% glycerol, 1.25 ml 1 M Tris pH 6.8, and 0.01 g bromophenol blue in 8 ml H_2_O) was added to the packed beads in a 1:1 ratio. Before using 2 × SB, 1 M DTT was added to a final concentration of 0.2 M. The sample with eluted protein as well as the previously prepared input sample were incubated for 25 min at 95 °C. All samples were then centrifuged for 1 min at 1800 g, at RT. The resulting supernatant was the 2 × eluted capture ready to use for immunoblotting procedure. Both the input sample and the purified iPOND capture sample were tested simultaneously.

### DNA fragmentation assay by agarose gel electrophoresis

To examine DNA fragmentation size after sonication, 90 μl of H_2_O and 4 μl of 5 M NaCl were added to 5 μl of lysate (control and after treatment with HU) and incubated overnight at 64 °C. Subsequently, samples were treated with 1 μl RNase A (20 mg/ml) for 30 min at 37 °C followed by Proteinase K (Sigma-Aldrich) treatment for 2 h at 45 °C (20 μl of 0.5 M EDTA, 40 μl of Tris pH 6.7 and 10 μl of Proteinase K). According to the procedure used earlier (Żabka et al. 2020), samples containing 10 μl of DNA and 2 μl of gel loading buffer (0.25% bromophenol blue, 30% glycerol in 1 × TAE composed of 40 mM Tris–HCl, 1 mM EDTA, 40 mM acetic acid, pH 8.0) were applied to 1.5% agarose gel in 1 × TAE buffer with ethidium bromide (1 mg/ml) and separated by electrophoresis at 75 V for 3 h at RT. UV-fluorescent DNA fragments were photographed using a Gel UV Slider (Phoretix 1D image store system; Phoretix, England) in the Laboratory of Microscopic Imaging and Specialized Biological Techniques at the Faculty of Biology and Environmental Protection (University of Lodz).

### Immunoblotting procedure

All proteins were analysed by Western blotting as described previously (Żabka et al. 2010). Proteins from input and capture samples were fractionated on 4–12% Bis Tris/2-(4-morpholino)-ethanesulfonic acid SDS-NuPAGE Novex gel and blotted onto polyvinylidene fluoride membrane (0.2-μm pore size). Post-translational histone modifications and histone H3 were detected using rabbit antibodies: anti-histone H3K56Ac (monoclonal), rabbit anti-histone H4K5Ac (polyclonal), rabbit anti-histone H3T45Ph (polyclonal), and rabbit anti-histone H3 antibodies (polyclonal) diluted to 1:500 using the Chromogenic Western Blot Immunodetection Kit (Invitrogen). Secondary goat anti-rabbit IgG antibody conjugated with alkaline phosphatase were used to detect primary antibodies. Band intensities of histone modifications were quantified by densitometry.

Immunofluorescence detections of acetylation of histone H4 on lysine 5 (H4K5Ac), acetylation of histone H3 on lysine 56 (H3K56Ac), and phosphorylation of histone H3 on threonine 45 (H3T45Ph).

Root meristems were fixed in 4% PBS-buffered paraformaldehyde for 20 min (20 °C). Cell nuclei were isolated, dropped onto slides (procedure according to Żabka et al. 2021a), and after drying, incubated in PBS-buffered 8% BSA and 4% Triton X-100 (Sigma-Aldrich) for 50 min (20 °C). Slides were then incubated with:

1. rabbit polyclonal anti-histone H4K5Ac antibodies (Sigma-Aldrich, dilution of 1:200);
2. rabbit monoclonal anti-histone H3K56Ac antibodies (Abcam, dilution of 1:200);
3. rabbit polyclonal anti-histone H3T45Ph antibodies (Abcam, dilution of 1:100).

The antibodies were dissolved in PBS containing 1% BSA. Incubations were conducted in the dark for 18 h at 4 °C. After washing with PBS, slides were incubated for 1.5 h (20 °C) with Alexa Fluor® 488-conjugated goat anti-rabbit secondary antibodies (1:400; Cell Signaling) counterstained with propidium iodide (PI; 0.3 mg mL-1; control series) or DAPI (HU series) and embedded in a mixture of PBS/glycerol (9:1) with 2.3% DABCO.

### AgNOR staining

AgNOR impregnation of the control cells was performed using procedure described by Żabka et al. (2021b). The seedlings were squeezed between two microscope slides to isolate chromosomes. The obtained suspension was dropped onto microscope slides, dried, and fixed with freshly prepared 3:1 methanol–acetic acid mixture (3:1, v/v). Slides were immersed in 2 × SSC at 60 °C for 45 min, rinsed in water, and dried. The freshly prepared staining solution consisting of 0.05% formic acid and 0.5 g of AgNO_3_ was dropped onto microscope slides (100 mL^−1^ per slide). After covering with a coverslip, slides were put in a wet chamber (80 °C for 3 min), washed with distilled water, dried, and mounted (Canada balsam).

### Observations and analyses

Fluorescence intensity analyses, the line plots and Interactive 3D Surface Plots were performed with the ImageJ software. Observations were made using E-600 epifluorescence microscope (Nikon) equipped with phase-contrast optics, U2 filter (UVB light; λ = 340–380 nm) for DAPI, G2 filter (green light; λ = 540/25 nm) for PI-stained cell nuclei, and B2 filter (blue light; λ = 465–496 nm) for Alexa Fluor® 488. Nuclear DNA fluorescence and quantitative analyses were made after converting colour images into greyscale images and expressed in arbitrary units as mean pixel value (pv) spanning the range from 0 (dark) to 255 (white). All data were expressed as the mean values ± standard deviation of the mean (± SD). Student’s *t*-tests for paired data were used to compare individual variables. All immunofluorescence analyses were repeated (at least three times).

Identification of individual cells in G1, S, G2 phases (for histone modifications) and in early-, mid-, and late-S phase cells (for EdU) was done, respectively, by microfluorimetric measurements of DNA content (following nuclear staining with DAPI or propidium iodide) and by evaluating of the type of nuclear labelling. Microfluorimetric analyses were carried out with the computerized cytometry (Cytofotometer v1.2 system) and calibrated in arbitrary units (a.u.).

## Results

### Replication stress (RS) generates chromosomal aberrations

**Fig. 1:**
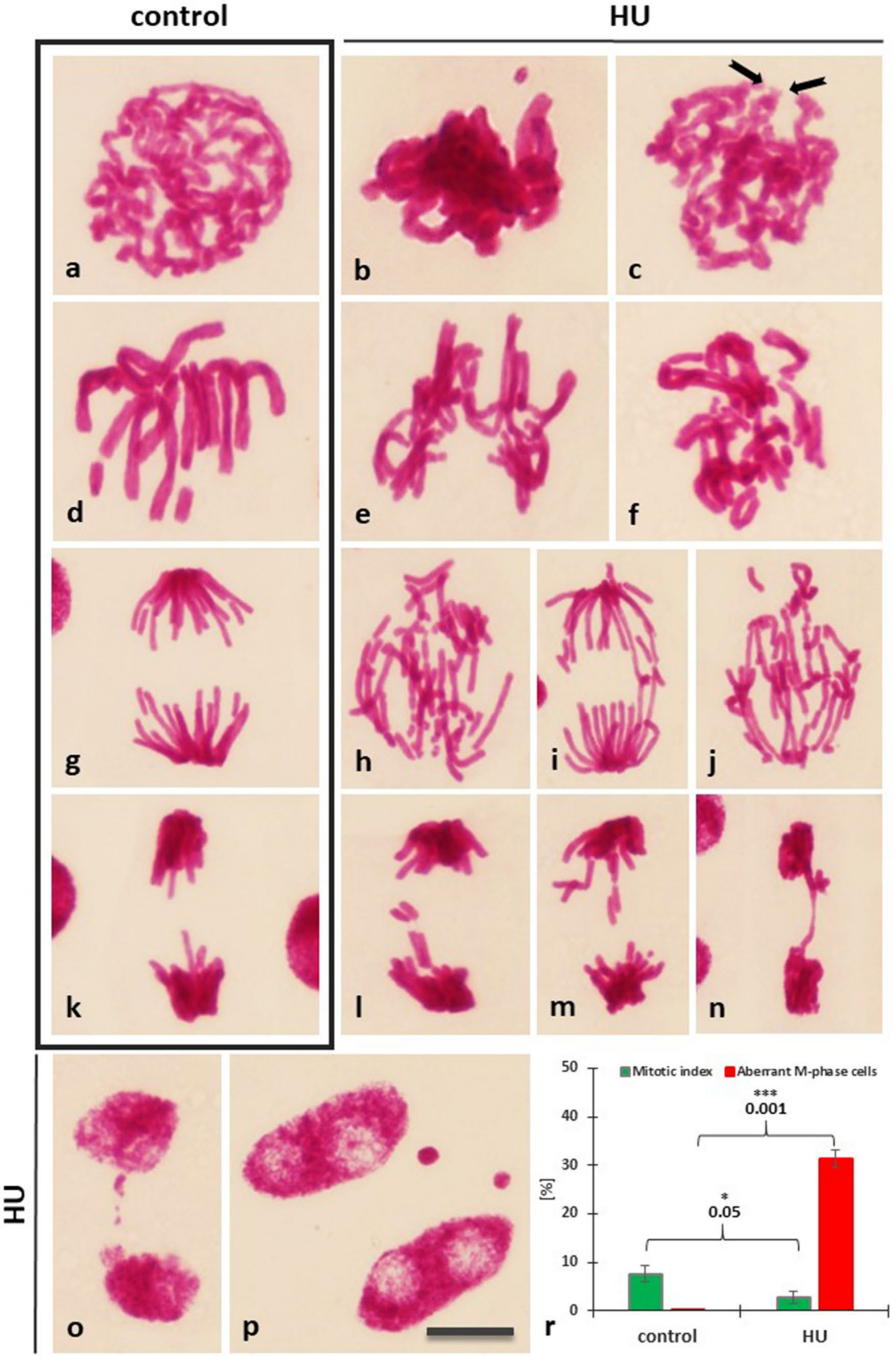
Feulgen DNA staining of *V. faba* cell nuclei from the control root meristems in **a** prophase, **d** metaphase, **g** anaphase, **k** telophase, and following 2-h treatment with 3 mM HU: (**b**, **c**) prophase, (**e**, **f**) metaphase, (**h–j**) anaphase, (**ln**) telophase. **o**, **p** Post-telophase cell nuclei with micronuclei formed after treatment with HU. Scale bar 10 μm. (*r*) Mitotic indices (% ± S.D.; green diagrams) and percentage of aberrant M-phase cells (% ± S.D.; red diagrams) in root meristem cells from *V. faba* seedlings after 2-h incubation with HU. Statistically significant change in MI values is marked by asterisks: * indicates *p* < 0.05 and *** indicates *p* < 0.001

Microscopic analyses of Feulgen stained *V. faba* root meristems treated for 2 h with 3 mM HU revealed a portion of mitotic cells with changes in chromosomal morphology (Fig. 1). Compared to the control seedlings displaying almost exclusively normal mitotic figures (Fig. 1a, d, g, k), 2 h treatment of HU revealed clustered chromosomes in prophase (Fig. 1b), only subtle breaks in the continuity of condensing chromatids (Fig. 1c) and a fraction of cells with chromosomes misaligned from the equatorial metaphase plate (Fig. 1e, f). Other structural aberrations, such as acentric chromosome fragments and chromosomal bridges, were observed in ana- (Fig. 1h–j) and telophase (Fig. 1l–n). Moreover, incubation with HU gave rise to extra-nuclear micronuclei containing lagging chromosomes or broken chromosome fragments that failed to incorporate into the nucleus after cell division (Fig. 1o–p). After 2 h HU treatment, bean root meristems revealed a threefold decrease in the relative number of mitotic cells, and the number of cells with aberrant chromatin morphology reached about 31% (Fig. 1r). Many of the chromosomal changes in *V. faba* resembled the PCC-like cells of *Allium cepa* created after prolonged treatment with low levels (0.75 mM) of HU (Żabka et al. 2012).

### HU-induced changes in DNA replication

**Fig. 2:**
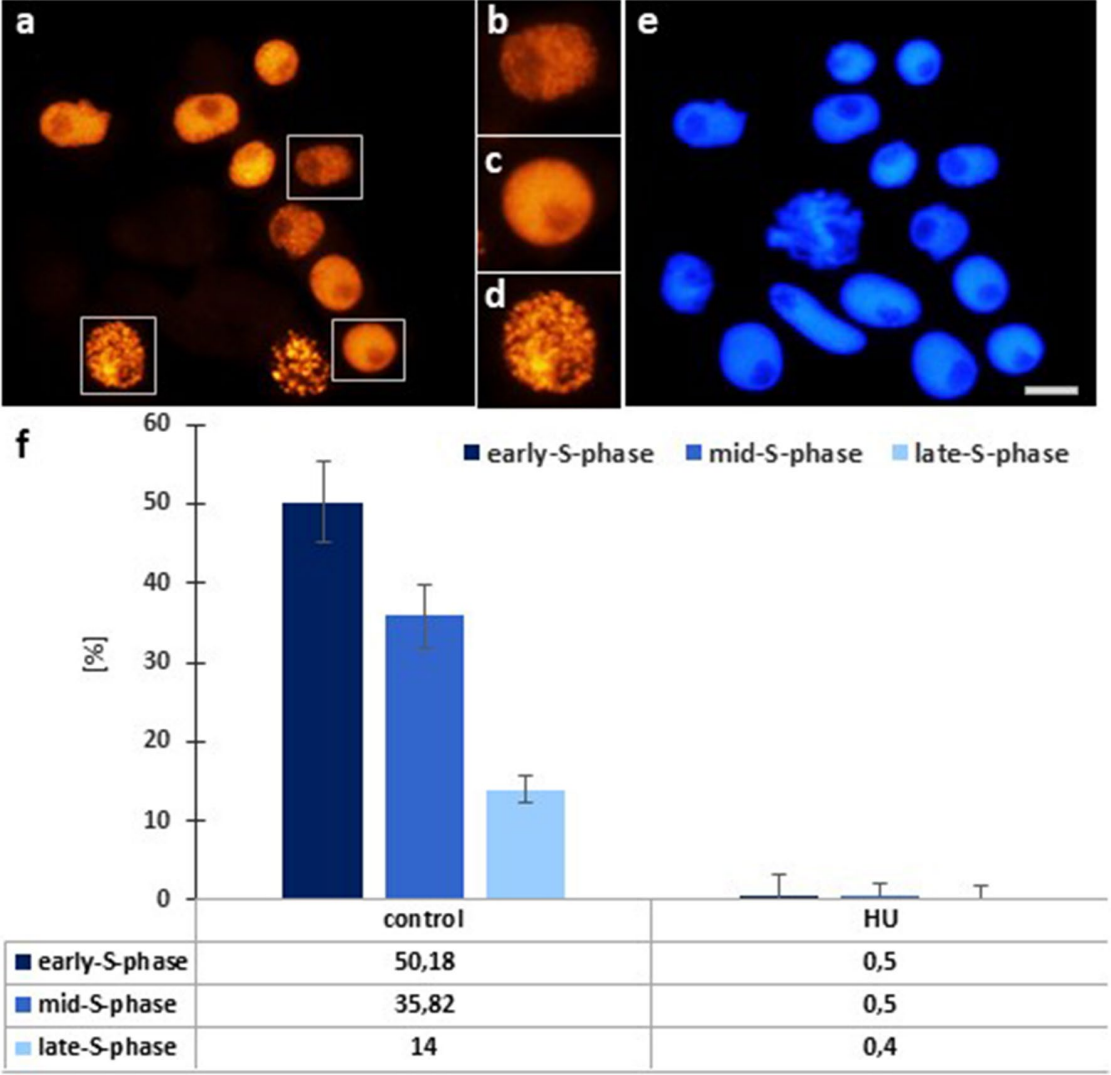
DNA replication in *V. faba* root cell populations evidenced by EdU incorporation. **a** Control cell population (boxes indicate cell nuclei shown at higher magnification), **b** early S-phase, **c** middle S-phase, **d** late S-phase; **e** cell population stained with DAPI corresponds with the population of EDU stained cells in Fig. 2a. Scale bar = 20 µm. **f** Fractions [(%) ± SD] of early-, mid-, and late-S-phase cells, calculated by combining microfluorimetric quantitation of DNA contents in DAPI-stained cell nuclei and visual analysis of EdU fluorescence patterns

The effect of a 2-h treatment with 3 mM HU on DNA replication was demonstrated by combining microfluorimetric quantification of DNA content in DAPI-stained cell nuclei (Fig. 2e) with EdU labeling analysis (Fig. 2a–d). Relative numbers of early-, mid-, and late-S phase cells were evaluated as the proportions of cell nuclei with weak (spotted), strong (almost homogeneous) and punctate (specific for heterochromatin) fluorescence staining patterns, respectively. The obtained data showed that the highest percentage of the control cells (about 50%) were in early-S-phase, and the lowest, in the late-S-phase (about 12%; Fig. 2f). HU drastically reduced all subpopulations of S-phase cells (to about 0.5%; Fig. 2f).

### Analysis of histone modifications using iPOND

Changes in the post-translational protein modifications at the RFs damaged by HU treatment compared with the RFs from the untreated *V. faba* root meristem cells were made using the iPOND method. The experiments included the impact of replication stress on: (i) histone H3 acetylation on lysine 56 (H3K56Ac), (ii) histone H4 acetylation on lysine 5 (H4K5Ac), (iii) histone H3 phosphorylation on threonine 45 (H3T45Ph), and (iv) histone H3, as a control.

**Fig. 3:**
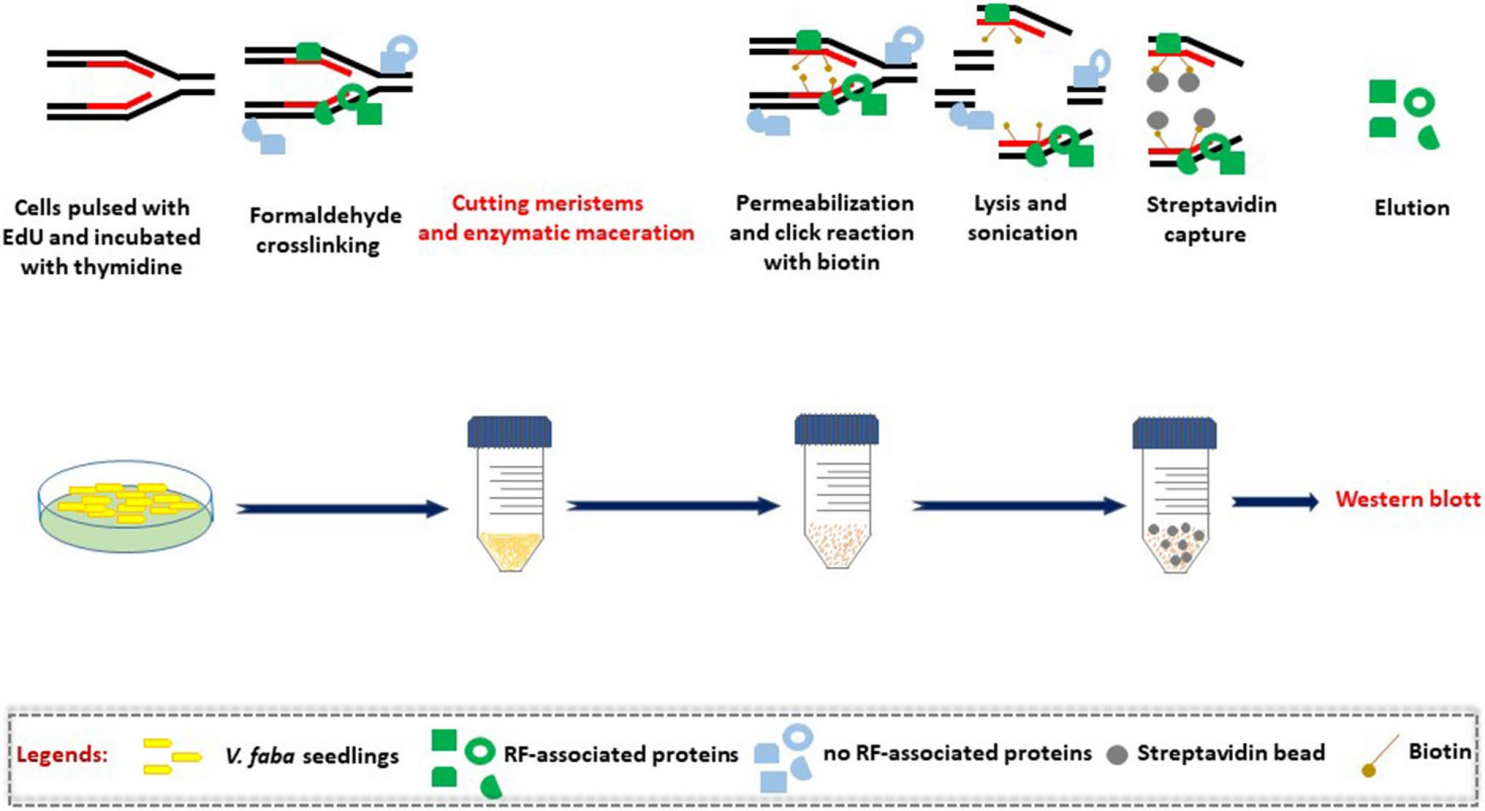
Graphical representation of the iPOND technique principles

Firstly, to mark newly replicated DNA, roots were incubated for 10 min with EdU, a thymidine analogue. After a brief (10 min) treatment with thymidine, part of the seedlings were incubated for 2 h in 3 mM HU and another part (control) with water (Fig. 3). After cross-linking of proteins and DNA using formaldehyde, meristems were cut off, cell walls were macerated, and a click reaction was performed to combine biotin with EdU. After cell lysis and DNA fragmentation (sonication), proteins close to biotin and EdU-labeled DNA were purified using streptavidin-coated agarose beads.

**Fig. 4:**
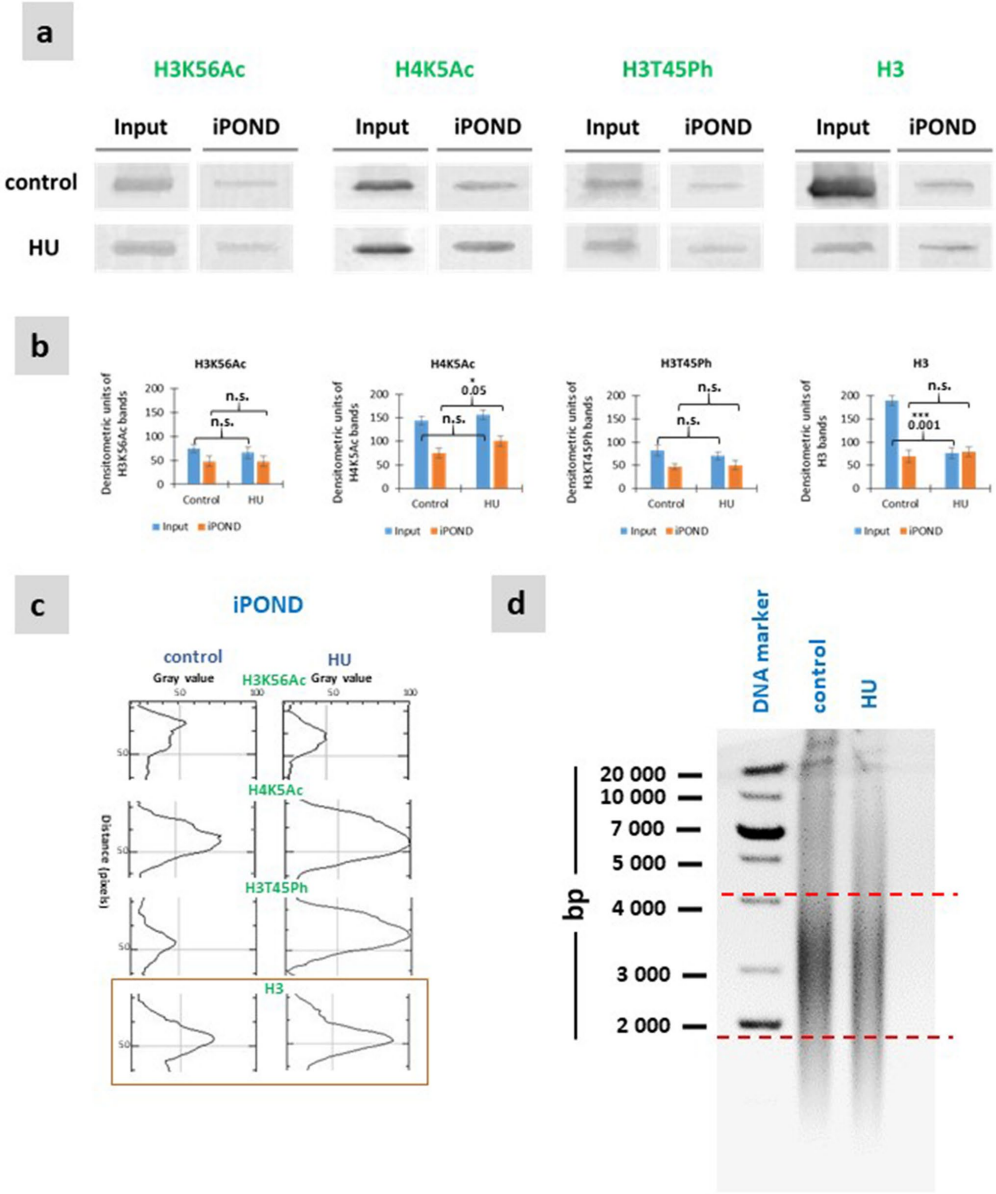
Immunoblot analysis and densitometry graphs showing post-translational histone modifications. **a** Input and EdU-associated proteins (iPOND) were analysed by Western blot using antibodies against H3K56Ac, H4K5Ac, H3T45Ph, and H3. **b** Densitometry plots showing the intensity of the bands (for the SDS-PAGE gel). **c** Densitometric plots of iPOND/Western blot bands for control and HU showing the ratio of histone modifications H3K56Ac, H4K5Ac, H3T45Ph to total histone H3. **d** DNA fragmentation detected with agarose gel electrophoresis of DNA extracted *V. faba* root meristem cells: lane 1—mass marker, lane 2—control, and lane 3—HU treatment for 2 h. Statistically significant changes (± SD) in the intensity of the bands (for the SDS-PAGE gel) (± SD) were assessed with Student’s t–test. The lack of significance is indicated by “n.s.” (not significant)

Both the input samples and the purified iPOND samples were tested using Western blot technique (Fig. 4a). Due to the selective effect of the iPOND procedure, all input samples (with the exception of the H3 histones obtained from HU-treated root cells) resulted in stronger bands (differences statistically significant). Immunoblotting analysis of iPOND-captured proteins combined with quantitative densitometric analyses showed that the amounts of H4K5Ac histones in HU-treated cells was increased, compared with the control series (Fig. 4a, b). In contrast to the above series, no significant differences were found in the quantities of iPOND-captured H3K56Ac, and H3T45Ph (Fig. 4b).

Densitometric plots of iPOND/Western blot bands showed that the levels of H3K56Ac and H3T45Ph in the control and H3K56Ac in the HU-treated samples were twofold lower compared to histone H3. Slightly higher values than those for total H3 were observed after HU treatment for H4K5Ac and H3T45Ph modifications (Fig. 4c).

In order to evaluate the extent of DNA fragmentation, the agarose gel electrophoresis was used (Fig. 4d). In both the control and HU-treated samples, the highest density of chromatin fragments ranged between 2000 and 4000 bp (Fig. 4d).

### Acetylation of histone H3 on lysine 56 (H3K56Ac)

**Fig. 5:**
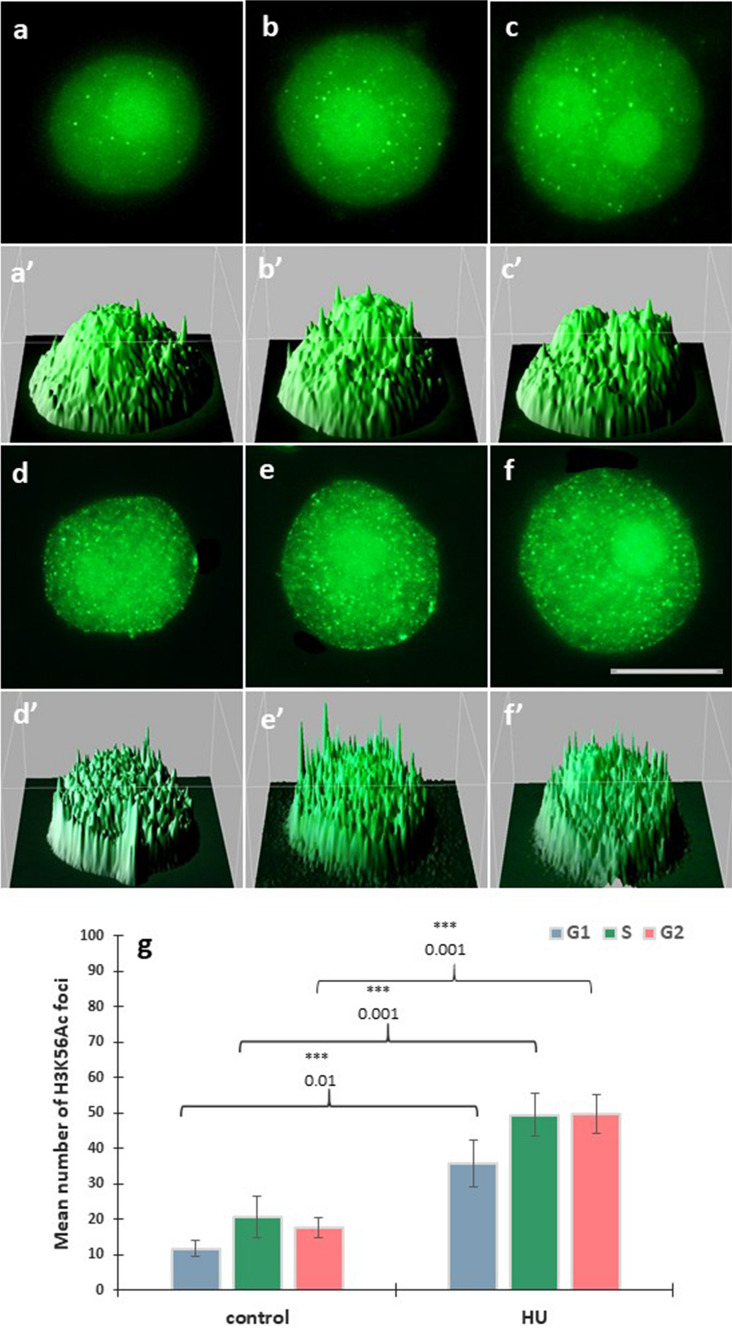
Immunofluorescence detection of H3K56Ac in cell nuclei from the control (**a–c**) and HU-treated root meristems (**d**–**f**) in the G1 (**a**, **d**), S (**b**, **e**), and G2 phases (**c**, **f**); scale bar = 10 μm. Below of each micrograph, corresponding interactive 3D surface plots (**a**’–**f**’). Mean number of H3K56Ac foci in G1, S, G2 phase nuclei in the control and HU-treated root meristem cells (**g**). Statistically significant changes in the intensity of H3K5Ac fluorescence (± SD) were assessed with Student’s *t*-test

HU-induced changes in histone 3 lysine-56 acetylation (H3K56Ac) were estimated by quantitation of the average number of intranuclear immunofluorescence foci, evidenced in G1, S and G2 phase cells (having 2, 2-4C and 4C DNA contents, respectively; Fig. 5a–f) by the Interactive 3D Surface Plot analysis (Fig. 5a’–f’). Both in the control and HU-treated root meristems, H3K56Ac foci were spread throughout the nuclear chromatin region (Fig. 5a–f) and localized at the border of the nucleolus and perinuclear chromatin (Fig. 5b, c). Incubation with HU brought about an increase in the average number of H3K56Ac histone foci at all stages of the cell cycle with the largest (nearly twofold) rise in the G1 and G2 phases of interphase (Fig. 5g).

### Acetylation of histone H4 on lysine 5 (H4K5Ac)

**Fig. 6:**
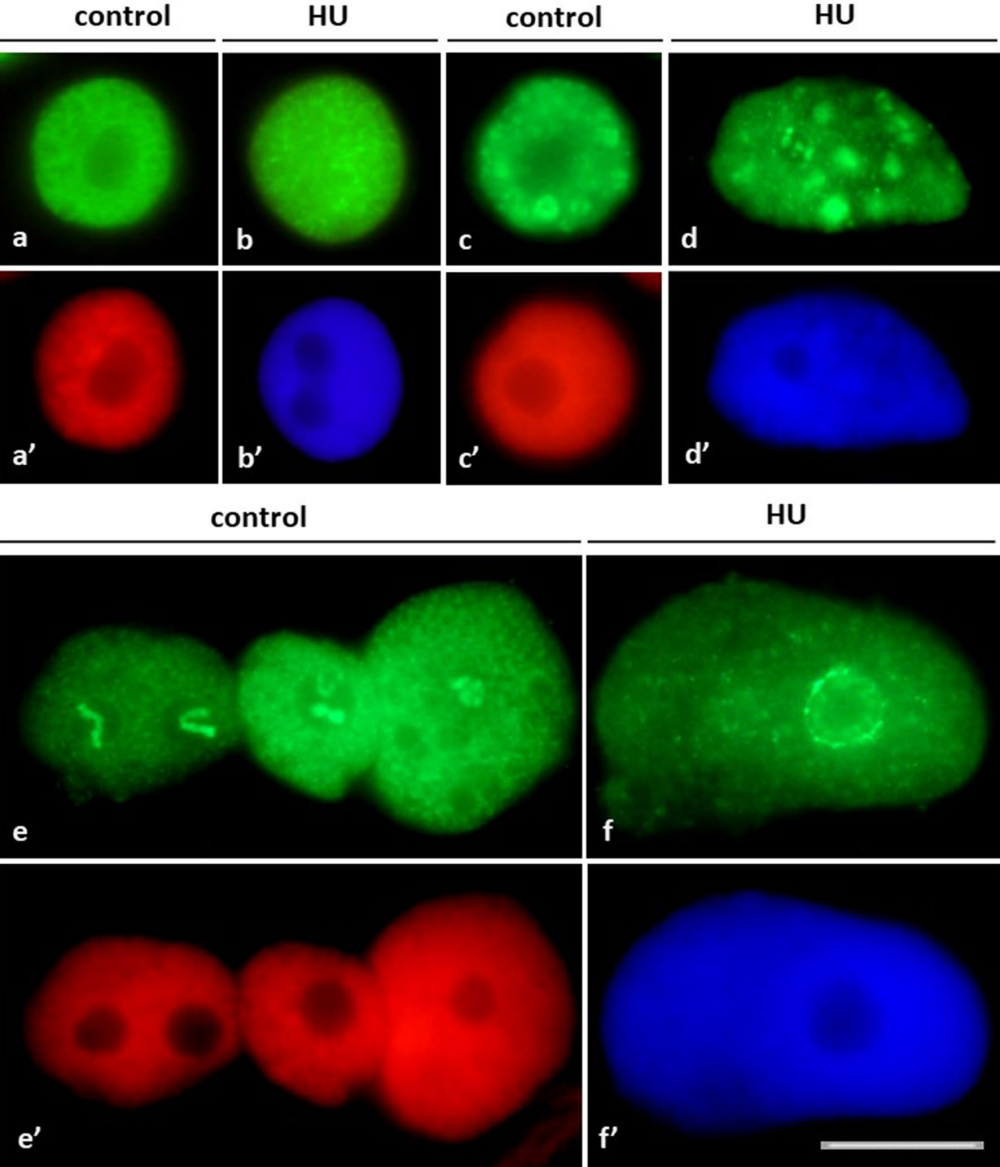
Intranuclear distribution of H4K5Ac immunofluorescence. **a**, **b** Cell nuclei with immunostained euchromatin areas: **a** control, **b** HU-treated cells and corresponding images of cell nuclei stained with propidium iodide (**a**’) and DAPI (**b**’). **c**, **d** Cell nuclei with immunostained heterochromatin areas: **c** control, **d** HU-treated cells and corresponding images of cell nuclei stained with propidium iodide (**c**’) and DAPI (**d**’). **e**–**f** Cell nuclei with nucleolar staining: **e** control, **f** HU-treated cells and corresponding images of cell nuclei stained with propidium iodide (**e**’) and DAPI (**f**’). Scale bar = 10 μm

**Fig. 7:**
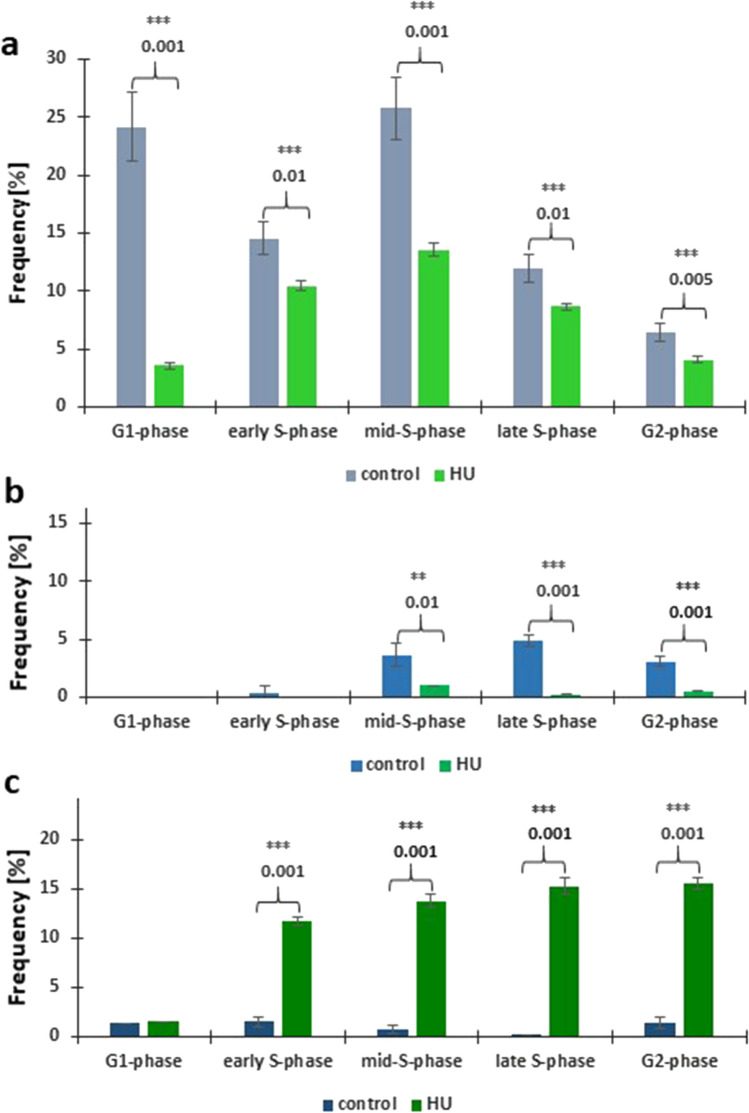
**a–c** Frequencies (% ± SD) of nuclei with H4K5Ac immunolabeling localized in: **a** euchromatic, **b** heterochromatic, **c** and nucleolar regions, discriminated with respect to successive stages of the cell cycle in root meristems. Statistically significant changes in the intensity of H4K5Ac fluorescence (± SD) were assessed with Student’s *t*-test

As compared to the previously described punctate (focal) distribution of chromatin regions enriched in lysine 56-acetylated H3 histones (H3K56Ac, characteristic of all types of nuclei both in the control and HU-treated root meristem cells in *V. faba*), immunofluorescence analysis of H4K5Ac revealed a considerably greater number of nuclear labeling patterns. The investigations performed throughout the whole cell cycle included quantitative estimation of: (1) cell nuclei with more or less homogeneous immunofluorescence staining, typical of euchromatin (Fig. 6a, b, a’–b’), (2) cell nuclei with large fluorescent areas of heterochromatin (Fig. 6c, d, c’, d’), and (3) cell nuclei with intensely stained nucleoli, mainly associated with weaker or stronger labeling of euchromatin (Fig. 6e, f, e’, f’). The performed analyses revealed that the relationships between the discerned nuclear areas enriched in H4K5Ac and different cell cycle stages are modified by HU-induced stress conditions, which is manifested by considerable changes in the proportions of cell nuclei with dominant immunofluorescence of euchromatin, heterochromatin, and/or nucleolar domains (Fig. 7a–c). Compared to the control population, a marked decrease in the intensity of H4K5Ac fluorescence in HU-treated root meristems was observed both in euchromatic-type nuclei (comprising mostly decondensed chromatin; Fig. 7a) and in the heterochromatic-type nuclei (comprising numerous clusters of condensed chromatin; Fig. 7b). Furthermore, at both G1 phase (in the control and HU-treated root meristems) and early S phase (in HU-treated plants), no cell nuclei were found with strongly stained condensed heterochromatin regions (Fig. 7b).

**Fig. 8:**
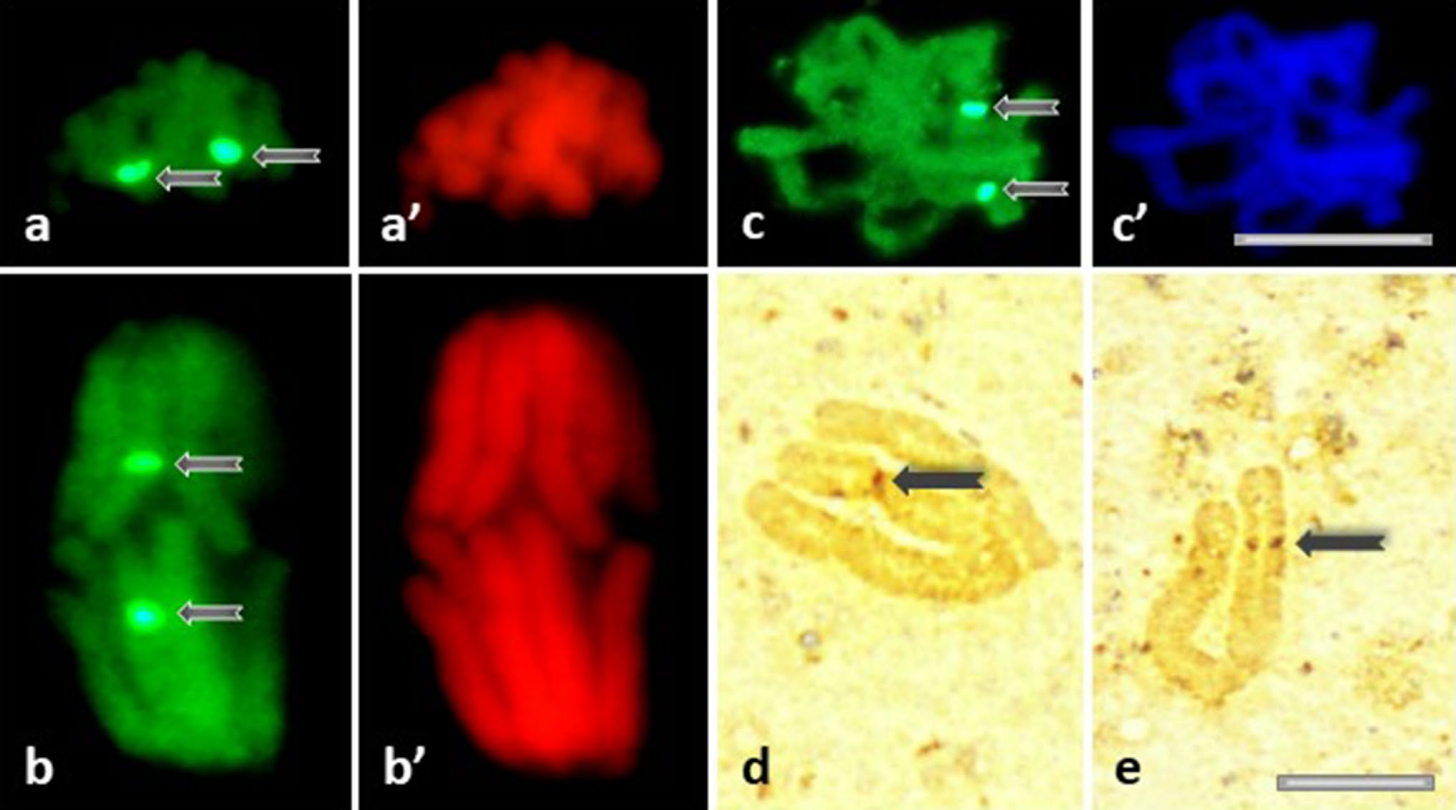
**a–c** H4K5Ac immunofluorescence labeling of the chromosomal areas corresponding to the nucleolus-organizing regions (NORs) in the control root meristems during: prometaphase (**a**), anaphase (**b**), and in the HU-treated cells during metaphase (**c**). The corresponding images of cell nuclei stained with propidium iodide (**a**’, **b**’) and DAPI (**c**’). **d**, **e** AgNOR staining of the control chromosomes: NOR-carrying isolated metaphase chromosomes. Arrows show NORs regions. Scale bar = 10 μm

In most cases, intense fluorescence of nucleoli was associated with more or less stained euchromatin background (Fig. 6e); moreover, the nucleoli were often surrounded by a circle of fluorescing dots (Fig. 6f). In contrast to eu- and heterochromatic-type nuclei described above, the population of cells with heavily stained nucleoli increased considerably in HU-treated *V. faba* root apical meristem (RAM) cells (starting from early S- up to G2 phase of the cell cycle; Fig. 7c). The fluorescence spots visible in prometaphase and anaphase (in the control, Fig. 8a, b)) and metaphase (after treatment with HU; Fig. 8c) correspond probably to brown chromosomal nucleolus-organizing regions (NORs) identified using silver staining procedure (AgNOR; Fig. 8d, e).

### Phosphorylation of histone H3 on threoninie 45 (H3T45Ph)

**Fig. 9:**
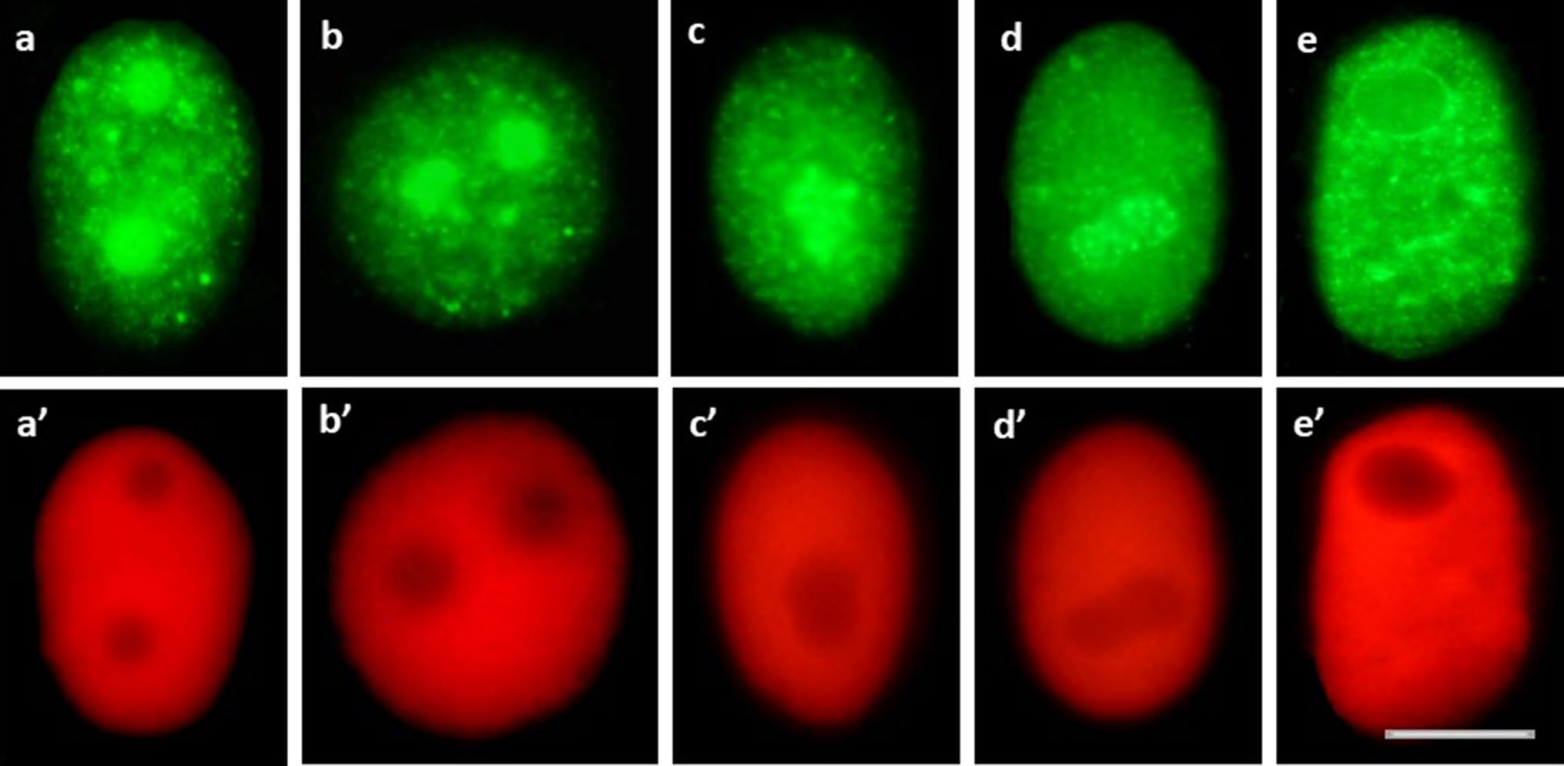
Different immunofluorescence patterns observed in root meristem cell nuclei using antibodies against H4T45Ph (**a–e**) and corresponding images of cell nuclei stained with propidium iodide (**a**’–**e**’). The control cells revealed predominant nucleolar staining of H4T45Ph (**a**–**d**) and specific labeling around the nucleoli (**e**). Scale bar = 10 μm

**Fig. 10:**
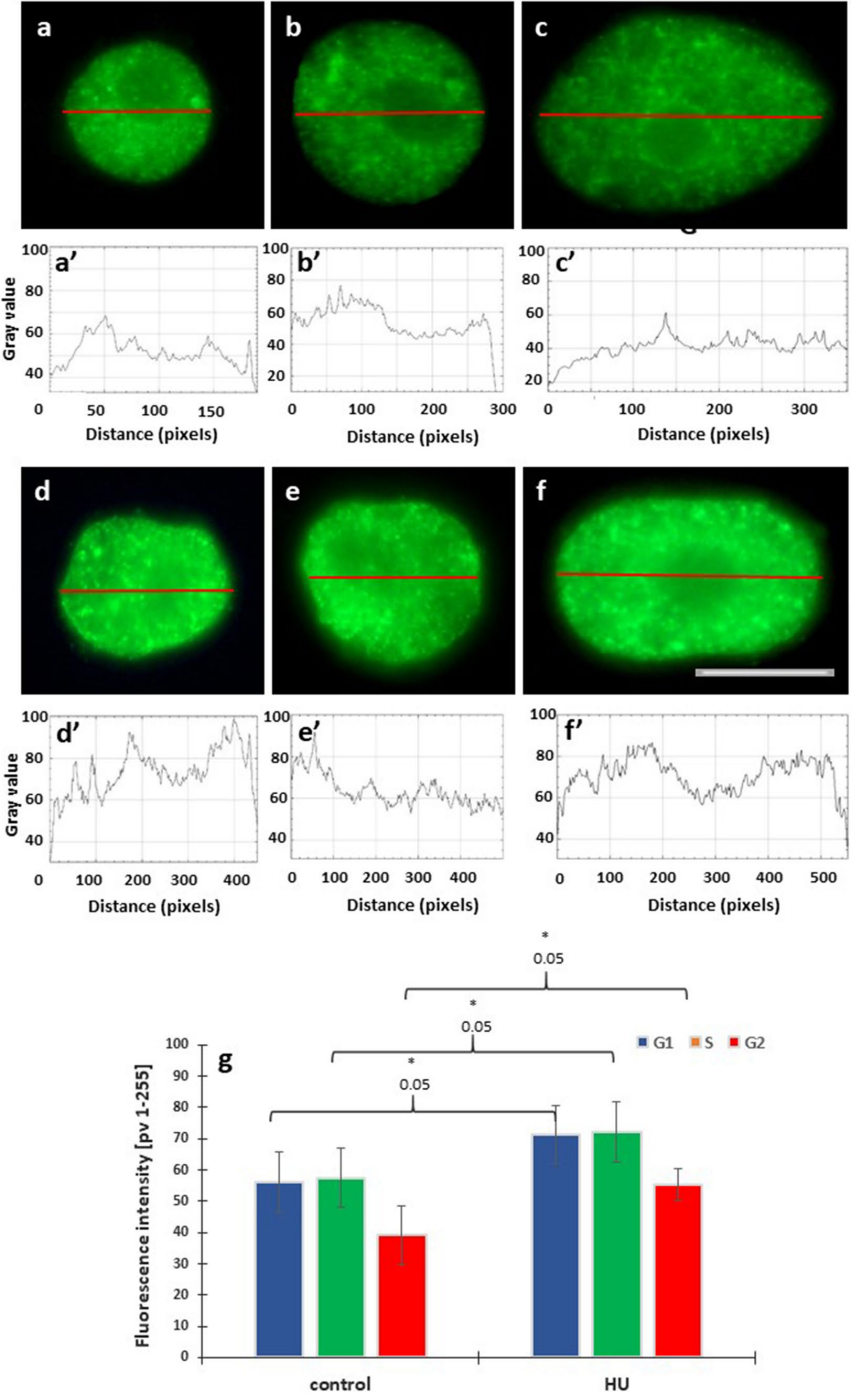
Immunofluorescence detection of H3T45Ph in cell nuclei from the control (**a**–**c**) and HU-treated seedlings of *V. faba* (**d**–**f**), in the G1 (**a**, **d**), S (**b**, **e**) and G2 phases (**c**, **f**); scale bar = 10 μm. Densitometric plots showing changes in fluorescence of the nuclear and nucleolus regions in the control (**a**’–**c**’) and HU-treated cells (**d**’–**f**’). Mean H3T45Ph fluorescence intensity of G1, S, G2 phase nuclei in the control and HU-treated meristem cells (**g**). Statistically significant changes in the intensity of H3K5Ac fluorescence (± SD) were assessed with Student’s *t*-test

Control and HU-treated root meristem cells labeled using anti-H3T45Ph immunofluorescence procedure displayed various nuclear and nucleolar staining patterns (Figs. 9, 10). Frequently, strong fluorescent labeling of nucleoli was accompanied by more or less intense fluorescence of the nuclear region (Figs. 9a–d, a’–d’), whereas weekly stained nucleoli were surrounded by a garland of immunofluorescent dots (Fig. 9e, e’).

Fluorescence intensity (FI) analysis of the whole nuclear areas in the G1, S, and G2 phase cells (Fig. 10), combined with line scans of fluorescence intensity (plots) through the nucleolar region revealed significantly higher levels of fluorescence in the nuclei of HU-treated meristematic cells (about 20% increase; Fig. 10a–c, a’–c’) than in the chromatin regions in the control cell nuclei (Fig. 10d–f, d’–f’). Both in the control and HU-treated root meristems, the FI values scored for the G2-phase cells were found significantly lower than that for the G1 and S phase cells.

## Discussion

The process of DNA replication requires both the dissociation of histones, separation of DNA strands and an integrated way of reassembling chromatin on the two DNA duplexes. Since both DNA replication and cellular response to replication stress (RS) occur in the context of chromatin, histone dynamics as well as functioning of proteins involved in the induction and progression of nuclear replication play a key roles in modulating replication forks (RFs) progression (Hsu et al. 2014; Zeman and Cimprich 2014). Prolonged disruption and/or inhibition of replication carries a high risk of altering newly formed chromatin in ways that can modify epigenetic information, affect the spatial organization of DNA and deregulate gene expression (Khurana and Oberdoerffer 2015).

Investigating the process of DNA replication, chromatin folding and maturation and, in particular, the response to RS is a major challenging task in the context of plant material. Our previous research on RS has focused around prolonged treatment of *Allium cepa* root meristems with low (0.75 mM) concentrations of HU or 5-aminouracil (5-AU), causing either premature chromosome condensation (PCC) or an abnormal organization of nuclear structures forming interphase and mitotic (IM) domains of chromatin at opposite poles of the IM cell nucleus (Żabka et al. 2010, 2012, 2015, 2020). We have shown that long-term incubation with 0.75 mM HU results in the raised levels of cyclin B-like proteins (Żabka et al. 2010) and increased production of reactive oxygen species (ROS), leading to DNA damage (manifested by γ-phosphorylation of histone H2AX and chromosomal aberrations; Żabka et al. 2012). We also provided evidence that there is an association between the opposite poles of IM cell nuclei and the polarized accumulation sites of auxin efflux carriers (PIN2 proteins) and IAA (auxins; Żabka et al. 2015). Furthermore, we have shown that continuous treatment of onion cells with 5-AU is linked with accelerated dynamics of the DNA replication machinery and significantly increased levels of transcription and translation (Żabka et al. 2020).

Since, more recently, our research has focused on RS and epigenetic modifications after cadmium treatment (Żabka et al. 2021a, b), we, therefore, began to search for a more sensitive method that would allow the analysis of post-translational histone modifications in active and inhibited or damaged RFs. Our attention and interest was drawn to a technique called iPOND (isolation of proteins on nascent DNA), first published in 2011 by Sirbu et al. (2011), which provides a high-resolution spatial–temporal analysis of proteins in RFs. This method, developed for animal cells, is used: (1) to identify proteins associated with active replisomes, (2) to monitor changes in chromatin located at different distances from RFs and, (3) to detect protein recruitment or post-translational modifications of proteins in damaged forks (Sirbu et al. 2011, 2012, 2013). The iPOND technique makes it possible to obtain proteins bound directly or indirectly to the nascent DNA. In this procedure, the nascent DNA is labeled with 5-ethynyl-2′-deoxyuridine (EdU; a nucleoside analogue of thymidine), which, via an alkyne group, enables copper-catalysed cycloaddition (click chemistry) to the biotin azide, ultimately yielding a stable covalent bond. This facilitates the purification of EdU-labeled DNA–protein complexes, using streptavidin-coated beads. After a final elution, the resulting proteins and their modifications can be analyzed by Western blot or mass spectrometry (Sirbu et al. 2011; Olcina et al. 2016; Sirbu et al. 2013).

While the complex role of chromatin in the DNA replication process has been studied for many years (Bellush and Whitehouse 2017), it is now thought that the key aspects of the RS response are also linked to chromatin. Our current research, conducted on *V. faba* root meristem cells, has focused on understanding issues related to changes in epigenetic patterns following short-term exposure to HU-induced RS. To achieve this, three histone modifications were investigated using the iPOND technique combined with immunocytochemical labeling: (i) acetylation of histone H3 at lysine 56 (H3K56Ac), (ii) acetylation of histone H4 at Lys 5 (H4K5Ac), (iii) phosphorylation of histone H3 at threonine 45 (H3T45Ph).

Adaptation of the iPOND technique to plant material required overcoming of several difficulties. The first and most important step was the selection of the material and the acquisition of sufficient quantities of cells. According to Sirbu et al. (2013), each research sample necessitates approximately 1 × 10^8^ of human cells for efficient capture of replicon proteins. The large number of cells needed for iPOND procedure is dictated by the sensitivity of the immunoblotting detection method. Taking into account a large nuclear DNA content (26.7 pg/2C nucleus) and a great number of cells per root meristem (about 12 115 ± 1295), we considered *V. faba* as an excellent material for the iPOND procedure. Another important modification was the enzymatic digestion of the cell walls. HU (3 mM) was used as a replication stress factor to arrest active replisomes and DNA synthesis (tested using EdU) and to facilitate the analysis of histone modifications with transiently or permanently arrested RFs.

Various post-translational modifications of histones, such as acetylation, methylation and phosphorylation, regulate many cellular processes, including gene transcription, DNA replication and repair (Goldberg et al. 2007; Han et al. 2007a, b; Li et al. 2007). Newly formed histone H3/H4 molecules undergo acetylation prior to their incorporation into chromatin. Acetylation of histone H3 on lysine 56 (H3K56Ac) is unique in that it is recognized as a marker of newly synthesized H3 molecules and occurs mainly during S-phase, suggesting that this modification is involved in DNA replication (Hans and Dimitrov 2001; Bej and Basak 2017; Mursalimov et al. 2019). Since H3K56Ac is also a key modification that occurs in response to DNA damage (Wang et al. 2008; Fu et al. 2013; Gates et al. 2017; Mursalimov et al. 2019) and its level increases during S-phase (Mursalimov et al. 2019), we monitored histone H3 acetylation on lysine 56 during the cell cycle progression. Using the iPOND method, a strong H3K56Ac band in both control and HU-treated cells was observed, clearly demonstrating the validity of the applied technique, but we did not find increased levels of H3K56 acetylation on newly deposited histones after HU treatment. In contrast, immunocytochemical studies showed that, compared to untreated material, HU-treated cells displayed a significant increase in the mean number of intranuclear foci, which is consistent with our previous studies using CdCl_2_-induced replication stress (Żabka et al. 2021a). Thus, the results obtained with these methods appear to be good indicators of DNA damage and support the theory of Masumoto et al. (Masumoto et al. 2005) that cells with DNA strand breaks maintain high levels of H3K56 acetylation and that the maintenance of this modification depends on DNA damage checkpoint proteins, creating favorable chromatin environment for DNA repair.

During replication, both ‘old’ and ‘new’ nucleosomes are deposited on each of the two DNA strands (Serra-Cardona and Zhang 2018). De novo synthesized histone H4 carries acetyl marks on lysines 5 and 12 (Grover et al. 2018). Using iPOND technique, we showed that acetylation of histone H4 on lysine 5 (H4K5ac), which is more associated with DNA replication than transcription (Jasencakova et al. 2000; Żabka et al. 2021a), was significantly increased in HU-treated plants, suggesting a strong connection with cell cycle arrest during S phase. It can be inferred that H4K5ac may be a key epigenetic modification in the regulation of cell cycle arrest (Wang et al. 2008) and may have an important regulatory role in DNA replication (Żabka et al. 2021b).

Our immunofluorescence experiments indicate that the distribution of histone H4K5Ac in untreated cell nuclei is highly dependent on both the cell cycle phase and the intranuclear localization within territories occupied by euchromatin, heterochromatin or nucleoli. It was shown that the 2 h HU treatment drastically reduced the distribution of H4K5Ac in the euchromatin region in all phases of the cell cycle (especially in G1-phase) and that there was no or negligible labeling of H4K5Ac in the heterochromatin region in G1-phase and early S-phase. Histone H4K5 acetylation plays an important regulatory role in both DNA replication and transcription (Jasencakova et al. 2001). Each of these processes requires transient unwinding of DNA strands, making them extremely susceptible to ROS-induced damage (Chan et al. 2012). In fact, irrespectively of the character of the inhibitory effect exerted by HU (either primary, via inhibition of RNR, or secondary, via excess of generated ROS) suppression of DNA metabolism may result in erosion of epigenetic markers observed at all stages of interphase. Such an effect would be similar to the decrease in histone H4K5 acetylation observed in *V. faba* RAM cells after treatment with cadmium (Żabka et al. 2021a). In contrast, the process of H4K5 acetylation in the nucleolar regions is less susceptible to HU, which, according to other authors (Sogo et al. 1984; Conconi et al. 1989, 1992; Dammann et al. 1993) can be explained by the fact that transcriptionally active rDNA genes are devoid of nucleosomes. In addition, both studies in barley (Idei et al. 1996) and our earlier experiments in bean (Żabka et al. 2021b) showed that the fluorescent spots visible in mitotic chromosomes topologically corresponded to NORs, as visualized using silver staining procedure (AgNOR). The specific pattern of H4K5 acetylation in NORs observed in *V. faba* root meristems, which does not correlate with either replication or transcriptional activity, seems explainable on the assumption of Jeppesen (et al. 1997). According to this concept, epigenetic markers established by histone acetylation may represent a mechanism for the propagation of ‘cellular memory’, whereby genes in chromatin regions active prior to mitotic division are marked by histone acetylation and, as a consequence, have the potential to be preferentially reactivated in the G1 phase of the new cell cycle. Acetylated H4 histones may then act as ‘markers’ for proteins required during the subsequent interphase to initiate early transcription and metabolic reactivation.

Phosphorylation of histone H3 on threonine 45 (H3T45Ph), like acetylation of histone H3 on lysine 56, is an important modification that occurs during DNA replication and is required for chromatin reassembly (Hyland et al. 2005; Baker et al. 2010). In the yeast *Saccharomyces cerevisiae*, this phosphorylation regulates many nuclear functions, including DNA damage repair, transcription, mitosis, apoptosis and sporulation (Ito et al. 2007; Hurd et al. 2009; Żabka et al. 2021a). Using the iPOND technique in *V. faba* root meristem cells, no increased levels of H3T45Ph phosphorylation have been found on newly deposited histones after HU treatment. In contrast, immunocytochemical studies showed that the 2 h incubation with HU resulted in a slight but significant increase in H3T45Ph fluorescence; a similar result was obtained for *S. cerevisiae* (Baker et al. 2010). This results may be consistent with the theory of Lee et al. (2015) that phosphorylation of H3T45 by protein kinase B (AKT) facilitates transcriptional activation of DNA damage-induced genes.

Despite extensive knowledge of RS and its negative effects in animals, the molecular aspects of RS induction in the plant cell world are still poorly understood. The iPOND method is an excellent proteomic tool to facilitate the identification and quantification of mechanisms involved in DNA replication, chromatin maturation and the response to RS (Cortez et al. 2017). This technique offers a wide range of possibilities, related to the determination of protein dynamics and posttranslational modifications in active, arrested and collapsed RFs (Żabka et al. 2010; Sirbu et al. 2011, 2012). Adoption of this method allows to identify chromatin-associated proteins and factors involved in RS. Certainly, the iPOND method may prove to be a key first step for a more in-depth understanding of the mechanism of DNA replication and cell’s response to RS in plants, and may bring new data on how histone modifications, coincide with the key aspects of the RS response.

## Data Availability

The datasets generated and/or analysed during the current study are available from the corresponding author on reasonable request.
